# Antioxidants in Fruit Fractions of Mediterranean Ancient Pear Cultivars

**DOI:** 10.3390/molecules28083559

**Published:** 2023-04-18

**Authors:** Giovanna Piluzza, Giuseppe Campesi, Guy D’hallewin, Maria Giovanna Molinu, Giovanni Antonio Re, Federico Sanna, Leonardo Sulas

**Affiliations:** 1 National Research Council, Institute for the Animal Production System in Mediterranean Environment, Traversa La Crucca 3, Località Baldinca, 07100 Sassari, Italy; giovanna.piluzza@cnr.it (G.P.); giuseppecampesi2003@gmail.com (G.C.); giovanniantonio.re@cnr.it (G.A.R.); federico.sanna@cnr.it (F.S.); leonardo.sulas@cnr.it (L.S.); 2 National Research Council, Institute of Sciences of Food Production, Traversa La Crucca 3, Località Baldinca, 07100 Sassari, Italy; guy.dhallewin@cnr.it

**Keywords:** rainfed environment, biodiversity, scattered fruit trees, extensive farming, peel, peduncle, core, flesh, bioactive compounds, chlorogenic acid, arbutin

## Abstract

Background: The genetic diversity of Sardinian pear germplasm has received limited attention regarding its chemical composition. Understanding this composition can aid in the setting up of resilient, extensive groves that offer multiple products and ecosystem services. This research aimed at investigating the antioxidant properties and phenolic compounds of ancient pear cultivars grown extensively in Sardinia (Italy); Methods: the cultivars Buttiru, Camusina, Spadona, and Coscia (as a reference) were compared. Fruit samples were manually peeled and cut. Their flesh, peel, core, and peduncle were frozen separately, lyophilized, and milled before being analysed; Results: The content of total phenolics (TotP), total flavonoids (TotF), condensed tannins (CT), and antioxidant capacity in each fruit part varied significantly among the cultivars. The TotP content was high in the peduncle (42.2–58.8 g GAE kg^−1^ DM) and low in flesh (6.4–17.7 g GAE kg^−1^ DM); Conclusions: the highest values of antioxidant capacity, TotP, NTP, TotF, and CT were found in the flesh of the cultivar Buttiru and in the peel of the cultivar Camusina. Chlorogenic acid was the major individual phenolic compound in peel, flesh and core, whereas arbutin was mostly present in the peduncle. Results can contribute to revise target exploitations of underutilized ancient pear cultivars.

## 1. Introduction

Fruit and vegetable phytochemicals play a crucial role in the prevention of chronic diseases, as obesity, diabetes, cardiovascular diseases, and cancer represent an emerging global health issue [1].

*Pyrus communis* L. is a typical crop of temperate climates and its fruit has numerous health benefits. These benefits are due to their diuretic, antitussive, and anti-inflammatory activities, which are useful to prevent several diseases [2]. Pear antioxidant, anti-inflammatory, and antiproliferative activities have been demonstrated in vitro [3], and they may improve the regulation of blood pressure and vascular function in middle-aged and older men, and in women with metabolic syndrome [4,5]. A metanalysis from Hu et al. [6] reported an inverse association between pear consumption and the risk of stroke. The compounds of the greatest interest for their antioxidant and functional properties include phenolic compounds. The pear is one of the oldest fruit crops that is largely consumed worldwide. It represents an important source of biologically active substances [3] and is an excellent source of soluble and insoluble fibres, which are essential for the gut health. The antioxidant phenolic compounds of pear fruit are mainly concentrated in the peel; they contribute to increase endogenous antioxidant systems, preventing the rise of free radicals and therefore offsetting oxidative stress [2].

Oxidative stress is associated with aging and some pathological situations such as cardiovascular disease, neurodegenerative syndromes, cancer, and obesity [7]. Additionally, soluble fibres feed the healthy bacteria in the gut. As such, they are considered prebiotics, which are associated with healthy aging and improved immunity [8].

The growing ecological awareness in the modern consumer has resulted in a greater demand for healthier foods. Recent studies carried out in Bosnia Herzegovina and Turkey have reported a significantly higher concentration of total phenolic compounds in autochthonous pears compared to commercial cultivars [9,10]. Phenolic acids, flavonoids, and glycosylated hydroquinone (mainly *β*-arbutin) are the main phenolic compounds in pear fruit, but considerable variations in quantitative composition depending on pedoclimatic, agronomic, and storage conditions have been recorded in different cultivars [11]. The *β*-arbutin, mainly concentrated in the peel, has been reported to be an effective antibiotic and skin whitening compound [8]. Chlorogenic acid (5-*O*-caffeoylquinic acid), the second most abundant phenolic compound in the flesh and peel has anti-inflammatory and antioxidant properties. Epicatechin is the predominant flavonoid present in the pear fruit, with different biological effects in vitro such as antioxidant, antimicrobial, anti-inflammatory, anticancer, and cardioprotective activity [8].

The island of Sardinia (Italy), located in the centre of Mediterranean basin, is reported as one of the five Blue Zones characterized by a high prevalence of long-living individuals, where life expectancy is higher than other places worldwide [12]. The longevity of the population is claimed to be related to various factors, such as a healthy and active lifestyle, based on a traditional diet, low in calories, and rich in plant-based phytonutrients (wholemeal bread, legumes, vegetables, and fruits) [7]. Due to the geographical isolation, the wide range of pedoclimatic conditions, and ancient traditions in agropastoral activities [13], Sardinia has a great plant diversity [14]. In particular, for the genus *Pyrus*, the long lasting anthropic and environmental pressure has singled out a great number of domesticated entities supplying a rich germplasm. Wild pears (mainly *Pyrus pyraster* L. and *P. amygdaliformis* Vill.) and their hybrids are spread all over the island. Traditionally, scattered trees along the roads, in cleared fields, and at the edges of farmland were trained and/or grafted with more productive and/or scaled ripening local varieties. The survival of this typical Sardinian agroecosystem has been warranted by grafting high positioned branches. This sustainable interaction between animal husbandry and crop production warrants pear fruit as both a food and feed source. In Mediterranean climates, incorporating pear fruit as a feed supplement for different classes of animals reared under extensive farming and rainfed conditions within agro-silvo-pastoral systems, is an essential practice [15]. The local cultivars, in fact, have a high variability for different agronomic traits such as fruit size, ripening time, soil adaptability, and resilience to environmental conditions, characterized by high temperatures and low water availability during the summer [16]. 

Unfortunately, in the past decades, genetic erosion of pear biodiversity has been observed, due to global trade determinants, aging of the rural population, and the progressive abandonment of farming practices [17]. The consumption of traditional pear varieties is at present relegated to local areas where it is still possible to find them. 

However, the recovery of neglected and underutilized but well-adapted fruit cultivars, and the protection of available biodiversity are currently relevant aspects given the growing attention to sustainable productions. 

Furthermore, the desired redesign of current farming systems into resilient and extensive groves can concurrently offer organic food, forage, pharmaceuticals, nutraceuticals, and multiple ecosystem services [18].

Old varieties have survived for thousands of years, adapting to changing and unpredictable climates and represent a very important source of genetic diversity in a new context altered by the effects of climate change. 

Most of the past studies on Sardinia pear biodiversity have been concerned with the pomological characteristics, aimed at establishing synonyms and/or homonyms and providing cultivar classification and registration. In addition, chemical analysis such as total soluble solids, dry matter, and fruit acidity were performed yearly on fruit collected near a randomized experimental orchard kept under standard management conditions [19,20]. 

The genetic diversity of domestic and wild Sardinian pear germplasm has been little studied so far and its chemical and functional composition is still unexplored. Undoubtedly, the characterization of native pear germplasm is a necessary step for the exploitation of its nutritional or other uses. To our knowledge, no study has previously reported on the Sardinian pear phytochemical composition nor on the composition in the different parts of fruit such as flesh, peel, core, and peduncle. We hypothesized that (i) pear fruit parts are featured by different phytochemical composition and (ii) pear fruits from ancient varieties grown as scattered trees are higher in bioactive compounds than a commercial variety from intensive orchards.

Therefore, the present research is aimed at investigating chemical and antioxidant properties of ancient pear cultivars grown extensively in Sardinia for their exploitation as a source of food, feed, and nutraceuticals. 

## 2. Results

### 2.1. Total Phenolic Content and Antioxidant Capacity

The content of total phenolics (TotP), non-tannic phenolics (NTP), tannic phenolics (TP), total flavonoids (TotF), condensed tannins (CT), and antioxidant capacity in each fruit part, namely flesh, peel, core, and peduncle, varied significantly among pear cultivars (Table 1, Table 2, Table 3 and Table 4). Overall, the flesh of Coscia and Spadona varieties had lower content in phenolics (TotP, NTP, TotF, and CT) and antioxidant capacity (Table 1). The Buttiru flesh showed the highest values, having approximately double the content of Trolox equivalent antioxidant capacity (TEAC, both ABTS and DPPH assay), TotP, TotF, and CT compared to Camusina.

In all analysed pear cultivars, the TotP content was high in the peduncle, ranging from 42.2 to 58.8 g gallic acid equivalent (GAE) kg^−1^ dry matter (DM), (Table 4), followed by the core 18.3–40.4 g GAE kg^−1^ DM (Table 3), peel 16.5–40.3 g GAE kg^−1^ DM (Table 2), and flesh 6.4–17.7 g GAE kg^−1^ DM (Table 1). The antioxidant capacity showed the same trend.

The peel of Camusina (Table 2) showed the highest antioxidant capacity with the ABTS assay (6.4 mmol 100 g^−1^ DM), TotP, NTP, and TotF values, followed by the Buttiru peel that exhibited the higher CT content (8.7 g delphinidin equivalent (DE) kg^−1^ DM)). It is worth nothing that the TotP, NTP, and TotF contents of Camusina peel were 4.5, 2.5, and 6-fold higher than the corresponding flesh values. The peel of Spadona and Coscia had approximately half of the TotP content than Camusina peel, less than half the antioxidant capacity, and about one third of the TotF content. In addition, the cultivars Spadona and Coscia had the lowest CT content.

In the cultivar Buttiru, the core distinctly exhibited the highest contents for all the analysed traits, whereas Spadona showed the lowest, except for NTP values (Table 3). For TotP, the core concentrations were in the following order Buttiru > Camusina > Coscia > Spadona, as well as TP, TotF, CT content, and antioxidant capacity.

The pear peduncle in the Camusina and Buttiru cultivars showed higher values for all parameters compared to Spadona and Coscia (Table 4). In addition, the cultivar Spadona had the lowest TP values compared with the cultivar Coscia.

Ranking all mean values of each analysed chemical class, reported in Table 1, Table 2, Table 3 and Table 4, it is possible to note that: for TotP, concentrations were in the following order: peduncle > core > peel > flesh, whereas for TotF peduncle > flesh > core > peel and for CT core> peduncle > flesh = peel. 

### 2.2. HPLC Analysis of Phenolic Compounds

Five individual phenolic compounds were identified and quantified in the fruit flesh, 14 compounds in the peel, 8 in the core, and 13 in the peduncle (Table 5, Table 6, Table 7 and Table 8). 

The following phenolic groups were detected in the pear fruit: hydroxybenzoic acid (gallic acid), hydroxycinnamic acid (chlorogenic acid, 3,5-Di-*O*-caffeoylquinic acid (3,5-DCQ) and isomers), glycosylated hydroquinone (arbutin), flavan-3-ols (epicatechin and catechin), and flavonol glycosides (quercetin 3-glucoside, quercetin 3-galattoside and quercetin 3-rutinoside, quercetin 3-*O*-(6″-*O*-malonyl)-β-glucoside, isorhamnetin 3-rutinoside and derivative) and flavanol. Molecules were identified by UV spectra and confirmed with authentic standards.

Comparing the examined fruit components, the flesh contained fewer phenolic compounds and in minor amounts. Chlorogenic acid was the most abundant phenolic compound in the flesh, ranging from 0.48 to 0.67 mg g^−1^ (Table 5). The cultivar Camusina showed the highest content. Arbutin was the second most abundant phenolic compound in the fruit flesh, found in all cultivars. The cultivar Camusina showed the highest content and the cultivar Buttiru showed the lowest. Flavonol glycosides and flavanols were not detected in the pear flesh. The highest sum of individual phenolics in the cultivar Camusina, compared to Buttiru, was not in agreement with the results obtained with Folin-Ciocalteu colorimetric assay. This discrepancy may be a clue of the presence of antioxidants other than phenolics in the Buttiru fruit flesh. Spadona was found to be the cultivar with the lowest content of individual phenolic compounds in the flesh.

Other than the individual compounds in the flesh, four additional molecules were identified in the peel, namely rutin (quercetin 3-rutinoside), quercetin 3-galattoside, quercetin 3-glucoside, and isorhamnetin 3-rutinoside. Based on the UV spectrum, related to a flavonol glycosides, other molecules were identified as four isorhamnetin derivatives and quantified as isorhamnetin 3-rutinoside equivalents (Table 6).

The results indicated that arbutin and chlorogenic acid were the main phenolic constituents in the peel of the analysed pear cultivars, followed by epicatechin and isorhamnetin 3-rutinoside. The cultivar Camusina showed higher content in almost all individual phenolic content detected, confirming the result obtained with the TotP. On the contrary, the cultivar Spadona showed lower contents, but it was the only cultivar having the quercetin 3-glucoside in its peel. The cultivar Coscia peel showed valuable contents of arbutin and chlorogenic acid. For individual phenolic compounds, peel concentration values were in the following order Camusina > Buttiru > Coscia > Spadona.

Compared to the peel, the core contained a Di-*O*-caffeolylquinic acid (t_R_ = 23.5) detected and quantified as 3,5-DCQ equivalents, having the same UV spectrum (Table 7). In addition, two other molecules, showing the flavanol spectra, were detected and quantified as epicatechin equivalents. No flavonol glycosides were detected in the fruit core.

In the core, arbutin and chlorogenic acid were the phenolic compounds with a higher content, followed by catechin and epicatechin. The cultivar Camusina core showed the highest content in chlorogenic acid and arbutin, whereas the cultivar Buttiru core evidenced the highest content in catechin and epicatechin (*p* ≤ 0.05). Furthermore, only in the cultivar Buttiru a flavanol t_R_ = 31.9 was present and in large quantities (4.91 mg g^−1^ DM), according to the highest total flavonoid content detected with the spectrophotometric assay (Table 3). The sum of individual phenolic contents was very similar in the cultivars Spadona and Coscia. However, their composition was different: the cultivar Coscia showed higher contents of arbutin, epicatechin, and catechin, whereas the cultivar Spadona was richer in chlorogenic acid.

A little content of 3,5-Di-*O*-caffeoylquinic acid was detected in the peduncle, compared to other fruit parts. In addition to the Di-*O*-caffeolylquinic acid (t_R_ = 23.5), another molecule with the same UV spectrum was detected in the peduncle (Table 8). This molecule, only found in the peduncle, was indicated as Di-*O*-caffeolylquinic acid (t_R_ = 24.7) and quantified as 3,5-DCQ equivalents.

Arbutin was the phenolic compound detected in all parts of the fruit, showing the highest concentration in the peduncle, where it was the most abundant molecule (7.49–5.11 mg g^−1^, Table 8) in the cultivars under study. The peduncles of Spadona and Camusina cultivars showed the significantly highest arbutin concentration followed by Coscia and Buttiru. In the peduncle of Spadona, the arbutin concentration was 9-, 16-, and 83-fold higher than in the core, peel, and flesh, respectively.

## 3. Discussion

Our study evaluated the content of total phenolics, individual phenolics, and the antioxidant capacity, in different fruit components of three ancient Sardinian pear cultivars that were selected based on their presence in different representative pedo-climatic areas of Sardinia, thus meeting the needs of most of the local agro-pastoral systems.

It is known that in the pear fruit the peel has higher phenolic contents than the flesh [10,21,22] which is also found in the cultivars under study. Pear samples purchased in a supermarket (without seeds and stalks, i.e., core) evidenced a content of total phenolic of 6.36 mg GAE g^−1^ [23], similar to our results for the Coscia flesh. 

The flesh of commercial fruits of five Australian pear cultivars (Rico, Packham’s Triumph, Beurre Bosc, Winter Nelis, and Josephine de Malines) evidenced a content of total phenolics and total flavonoids from 1.89 to 3.14 mg g^−1^ and from 0.57 to 1.53, respectively [24], which are lower than our results. It is worth noting that not only the cultivars were different, but the samples were extracted with a different solvent and solvent to solute ratio. Another Australian study on different pear cultivars (Packhams’s, Triumph) [25], showed in their peel a content of TotP and TotF of 4.30 mg GAE g^−1^ and 1.07 mg QE g^−1^ respectively again lower than our results.

Jiang et al. [26] showed that the phenolic content and antioxidant capacity of four pear cultivars had a decreasing trend throughout the developmental stages. The study was performed without partitioning into fruit components (peel or flesh); our results for flesh are within the range indicated by the authors. 

Within the Rosaceae family, peaches and plums are also rich in phenolic compounds [27]. As reviewed by Decros et al. [28], these metabolites exert antioxidant activity on both humans (after fruit consumption) and fruits to keep their redox homeostasis. Although other fruits can accumulate more secondary metabolites and display higher antioxidant activity than pears or apples, the widespread consumption of fruits from Rosaceae family makes them an important source of antioxidant compounds [27]. The chemical characterization of peach, pear, and plum, all belonging to the Rosaceae family, evidenced a total phenolics content of 0.43, 0.29, and 0.62 mg GAE g^−1^ FW, respectively [27]. These values were lower than our results, but they performed the experiment on fresh samples, with a different solvent and extraction method. In the peel of apple cultivars from Germany, Koschonsek et al. [29] found a total phenolic content that ranged from 521.9 to 1590.5 mg/100 g and an antioxidant capacity from 2.4 to 12.8 mmol TE/100 g DM. In the flesh of the same apple cultivars the total phenolic ranged from 143.6 to 361.7 mg/100 g and the antioxidant capacity varied from 0.8 to 2.3 mmol TE/100 g DM. Therefore, overall higher values of antioxidants were found in the apple peel than in the flesh in our results for the same fruit components.

The peduncle is a non-edible part of the pear fruit, whereas the core is edible, but being cartilaginous and difficult to digest, it is generally discarded together with the seeds. Conversely, grazing animals can eat the whole fruit from scattered trees, so these fruit components constitute a good source of phenolic compounds as feed. 

Moreover, the peel, core, and peduncle are bio-residue components resulting in pear juice-processing; therefore, as by-products from the agro-food industry, they can be a source of phytochemicals and antioxidants, which can be extracted for numerous applications: food additives, nutraceuticals, or ingredients in cosmetic products [30].

The structural properties and monosaccharides component of dietary fibre in pear pomace were analysed [31,32,33,34]. In the literature there is little information regarding phenolic compounds in pear pomace. A study demonstrated that the content of phenolic compounds, flavonoids, catechins, phenolic acids, and antioxidant activities was higher in the peel and pomace of the Khechechuri pear, native from Western Georgia, compared with the pulp and juice [35]. 

Lomba-Viana [36] reported a total phenol content of 29.35 mg GAE 100 g^−1^ and a DPPH value of 3.62 µm TE g^−1^ in the pomace of the pear Rocha cultivar, consisting mainly of peel and core. These values were lower than our results with respect to the peel, core, and pulp. In our study, the high amount of phenolics detected in the by-products (i.e., peel, core, and peduncle of ancient pear cultivars) may be related to the rootstock. In fact, the rootstock metabolizes high levels of secondary metabolites that are translocated to the fruit and stored into the fruit, especially in parts that are considered by-products or waste [37]. Huge quantities of by-products/wastes generated in fruit or vegetable processing are often discarded, whereas the utilization of bioactive components from by-products can improve the economic feasibility of the processing industry and help reduce the environmental pollution [38].

For example, onion peel and skin of whole yellow onions cultivated in Sweden had total phenolics content ranging from 27.8 to 51.1 and 54.7 to 68.2 mg GAE g^−1^ DM, respectively. A higher total polyphenolic compound content (97.28 mg GAE/g DW) paired with a valuable content of flavonoids (55.27 mg QE/g DW) was reported in the peel of yellow onions from Romania [38]. The above-mentioned values of phenolics compounds and flavonoids were higher than our pear values. 

The total phenolics and antioxidant capacity of testa oils from six genotypes of *Cocos nucifera* varied from 6.84 to 8.67 mg GAE/100 g and 15.89 to 31.95 (μmol TE/g), respectively [39] which is lower than our results.

Pedo-climatic factors, intended as abiotic stressors, influence secondary metabolism. Thus, levels and characteristics of metabolites, such as polyphenols, are highly variable according to the environment. Since the fruits used for our study were harvested from un-trained trees, we may assume that they were relatively rich in secondary metabolites, such as antioxidants. This statement is supported by Ref. [40] and is a positive issue for the dissemination of this innovative in situ preservation of pear biodiversity. In addition, compared to other rootstocks, wild pear ones are highly resistant to both biotic and abiotic stresses contributing to the build-up of a secondary metabolite sink in the fruit. As reported previously in the literature [10,41,42], individual phenolic compounds are distinctive for each cultivar, and differed between different fruit parts, both for quality and quantity. The greatest quantity of pear phytonutrients is concentrated in the peel rather than in the pulp, as reported by several authors [10,42,43,44,45,46,47].

Our findings regarding the chlorogenic acid content in the flesh are consistent with those obtained by Öztürk et al. [10], which reported a content of chlorogenic acid from 15.8 to 891.9 mg kg^−1^ in 13 local and 4 international pear cultivars grown in Turkey. The wide range was dependent solely on varietal characteristics as environmental and agronomic conditions were the same for all cultivars. A great variation in the content of chlorogenic acid was also observed by Akagić et al. [9], who studied Bosnian cultivars from a collection orchard. During two growing years, the chlorogenic acid in the flesh ranged from 0.46 to 14.94 mg kg^−1^ (first year) and from 0.10 to 9.69 mg kg^−1^ (second year), and the values were lower than our data. Similar to our results, the same authors did not detect the flavonol glycoside in the flesh, whereas Öztürk et al. [10] found p-coumaric and caffeic acids that were not detected in our study. The arbutin, gallic acid, chlorogenic acid, catechin, and epicatechin were found in five Australian cultivars as our results, with great variations among cultivars [24]. The same authors also detected quercetin, kaempferol, and caffeic acid; conversely, these compounds were not detected in our study. 

Again, the arbutin, epicatechin, and chlorogenic acid were the major phenolic compounds found in the pear skin (i.e., peel), which is in line with our results, whereas arbutin was the highest in the pulp [48]. Galvis-Sanchez et al. [49] found arbutin only in the pear skin, whereas we found this compound both in the flesh and peel, but with a higher content in the flesh. Chlorogenic acid is a bioactive compound with anti-inflammatory and antioxidant activity [8]. Arbutin has a tyrosinase inhibitory activity and interrupts melanin biosynthesis in epidermal cells; it is an active ingredient in skincare and cosmetic products as a skin-lightening agent. Arbutin also has antibacterial properties and is commonly used in the treatment of urinary tract infections.

Brahem et al. [41] identified the quercetin 3-rutinoside, quercetin 3-galattoside, quercetin 3-glucoside, and isorhamnetin 3-rutinoside using a HPLC/ESI-MS2, with the same elution order detected in our study. In addition, they identified six other molecules as two isorhamnetin-3-*O*-rutinosides, two iso-rhamnetin-3-*O*-hexosides, and two isorhamnetin acetyl hexosides, eluting sequentially as in our experiment. Based on their results, four molecules, with the same UV spectrum relative to a flavonol glycoside, were identified as isorhamnetin derivatives. Similar results were described by Kolniak-Ostek and Oszmiański [42], who identified isorhamnetin 3-*O*-rutinoside, galactoside, and glucoside and two isorhamnetin-acylated-hexosides, in pear fruits and leaves. A standardized method based on liquid chromatography with diode array and electrospray ionization/mass spectrometric detection (LC-DAD-ESI/MS) was used to analyse the phenolic compounds in the skin of 16 pear commercial cultivars [45]. The individual phenolic compounds that we detected in the peel were also found in this study. Öztürk et al. [10] found a content of chlorogenic acid from 21 to 1348.4 mg kg^−1^ in the peel; the highest values reported in this study were comparable to our results. Akagić et al. [9] reported a content of chlorogenic acid from 2.27 to 359.96 mg kg^−1^ in the peel during two years of observations which is lower than our results. Liaudanskas et al. [23] detected and quantified the quercetin 3-*O*-(6″-*O*-malonyl)-β-glucoside in the pear samples of different popular cultivars namely Conference, Concordia, Grabova, and Patten. Presumably, they found a content lower than our results because they analysed the entire fruit without distinction between the peel and the flesh. Lin et al. [45] also detected the quercetin 3-*O*-(6″-*O*-malonyl)-β-glucoside in the peel of *P. communis* varieties.

Gallic acid was found in peach and pear but not in plum, whereas the chlorogenic acid, catechin, epicatechin, and quercetin 3-galattoside were found in the three species, [27]. These phenolic compounds were also found in our study but at higher values; however, we performed the experiment with freeze dried samples and a different extraction method. Koschonsek et al. [29] detected gallic acid, chlorogenic acid, catechin, epicatechin, quercetin 3-galattoside, and rutin in the apple peel, and we detected the same phenolic compounds in the pear peel.

Concerning the core composition, a Di-*O*-caffeolylquinic acid was identified at a t_R_ of 23.5 min, eluting after the isorhamnetin 3-rutinoside (t_R_ = 23.09); Kolniak-Ostek [47] in the pear peel and flesh, and detected the 3–4-Di-*O*-caffeolylquinic acid after isorhamnetin 3-rutinoside, which is in line with our results for the core. Similarly, Brahem et al. [41] identified, in the flesh of polish pears, a Di-*O*-caffeoylquinic acid, eluting near the isorhamnetin-3-*O*-rutinoside, but they did not explain the chemical structure. In the three Sardinian cultivars, the very high concentration of arbutin was the distinctive trait of pear peduncles. Interestingly, the antioxidant capacity of peduncles showed higher values of ABTS than DPPH in all analysed cultivars; this trend was not found in the other parts of the fruit. Our result was confirmed by Takebayashi et al. [50], who showed that arbutin was a weaker scavenger against DPPH radical, but a more potent scavenger against ABTS cation radical.

## 4. Materials and Methods

The pear fruit identification and harvesting were carried out in Sardinia (Italy), 40° N, 8° E, 200 m a.s.l., where the climate is Mediterranean with a mild winter. The location has a subacid, sandy clay loam (Eutric, Mollic Fluvisols), with a long-term average annual rainfall of 580 mm and a mean annual air temperature of 16.6 °C. In this area, pear cultivars have been traditionally grown as scattered trees within an agroforestry context dominated by Mediterranean silvo-pastoral systems.

### 4.1. Plant Material and Fruit Sampling

**Figure 1:**
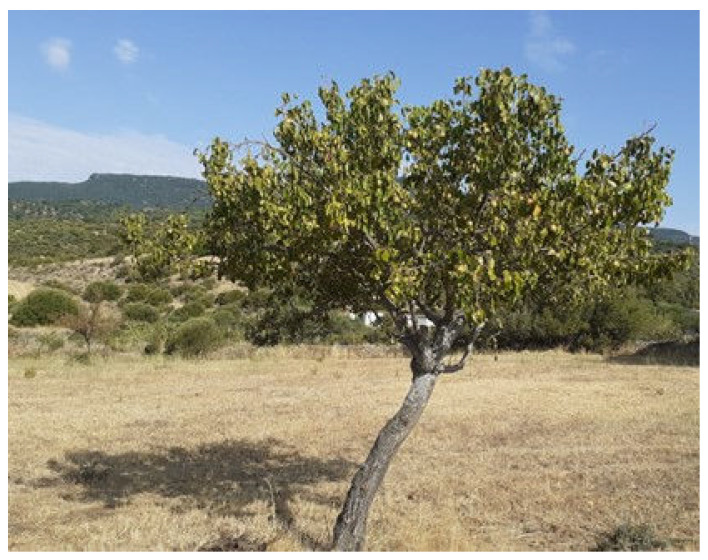
Scattered tree of the cultivar Buttiru in a typical rainfed Mediterranean environment in early September.

**Figure 2:**
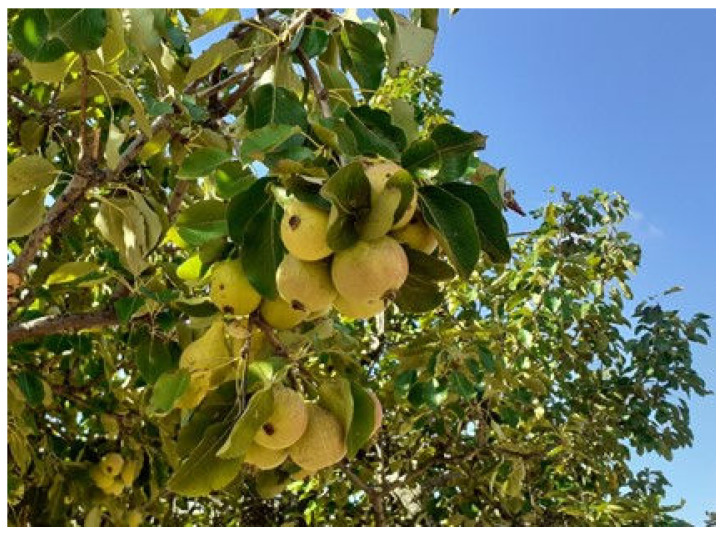
Details of the fruits of Camusina cultivar to be harvested in early August.

**Figure 3:**
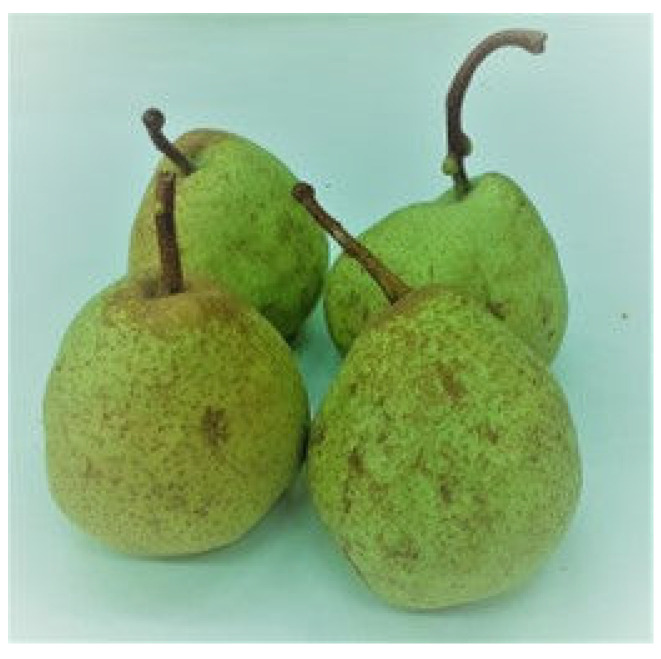
Fruits of Spadona cultivar to be harvested in early October.

From three ancient pear cultivars, namely Buttiru, Camusina, Spadona [51], fruits were collected at commercial maturity in August, September, October 2021, when relative humidity was 57, 70, and 75%, respectively. As a control, the Italian Coscia cultivar was purchased in a supermarket. The choice of these three ancient cultivars (Figure 1, Figure 2 and Figure 3) is based on their presence in all representative pedo-climatic and agro-ecosystems of Sardinia, which warrants a greater significance of the results. In addition, with the aim to extend the feeding period of the cultivars, we selected a sequential ripening base. The Italian Coscia cultivar is very common in the market, produced by intensive farming and present in most fruit producing areas of Sardinia.

**Figure 4:**
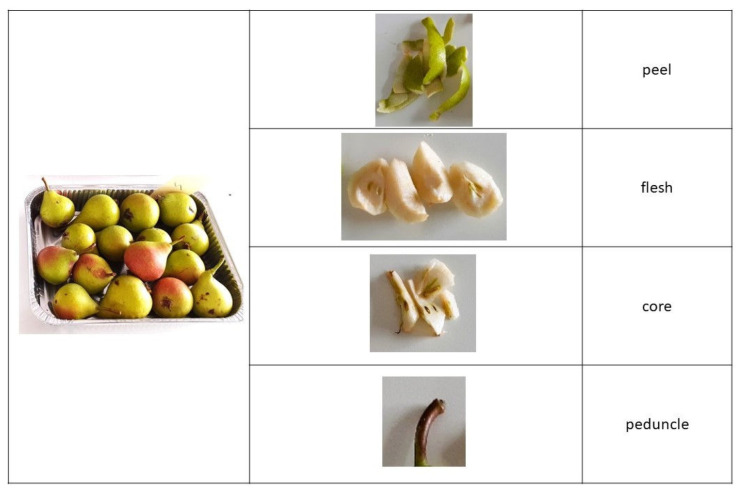
Fresh fruit partitioning into the four components for subsample preparation and storage before chemical analysis.

Fruits, free of defects and mechanical damage, were selected and each subsample was constituted by 10 homogeneous pear fruits replicated 3 times. They were manually peeled, and four wedges were cut vertically from each side. The flesh, the peel, the core (without seeds), and the peduncle (Figure 4) were frozen separately at −20 °C, before being freeze-dried using a Heto Lyolab 3000 (Heto-Holten A/S, Allerød, Denmark) for 80 h (−55 °C). After lyophilization, the samples were ground in a mill to a fine powder and stored in total darkness at −20 °C until further analyses. Sample extract preparation procedures were performed according to Molinu et al. [52], with some modifications. Briefly, 250 mg of the lyophilized sample was extracted with a 3 mL methanol/water (80:20 *v/v*) mixture and subjected to shaking for 24 h in the dark. Homogenates were centrifuged (10 min at 3900 rpm), and the supernatant was filtered using 0.20 μm polytetrafluoroethylene syringe filters and stored at −20 °C before further analysis. Methanolic extracts were then analysed for antioxidant activity, total phenolics, non-tannic phenolics, tannic phenolics, condensed tannins, and individual phenolic compounds. 

### 4.2. Total Phenolic Content and Antioxidant Capacity

Total phenolics (TotP), non-tannic phenolics (NTP), and tannic phenolics (TP) of ex-tracts were evaluated using spectrophotometric analysis with the Folin–Ciocalteau reagent, according to procedures previously described [53]. The results were expressed as g of gallic acid equivalent (GAE)·kg^−1^ dry matter. Total flavonoids (TotF) were quantified by colorimetric assay using the AlCl_3_ method, following procedures previously reported [53] and the results were expressed as g of catechin equivalent (CE)·kg^−1^ dry matter. 

The butanol assay was used for quantification of the extractable condensed tannin content from the sample, expressed as g delphinidin equivalent (DE)·kg^−1^ DM [54].

Antioxidant capacity was evaluated by means of the ABTS ((2,2′-azinobis (3-ethylbenzothiazoline-6-sulfonic acid) diammonium salt)) and DPPH (1,1-diphenyl-2-picrylhydrazyl) assays, as reported by Sanna et al. [53]. The results were expressed in terms of Trolox equivalent antioxidant capacity (TEAC), as mmol Trolox equivalents·100 g^−1^ dry matter (mmol TEAC·100 g^−1^ DM).

### 4.3. Reverse Phase-High-Performance Liquid Chromatography (RP-HPLC) Analysis of Phenolic Compounds

Chromatographic separation was carried out with the RP-HPLC method using an Agilent 1260 series HPLC instrument (Agilent Technologies, Palo Alto, CA, USA) equipped with a quaternary pump (G1311B), degasser, column thermostat (G1316A), auto-sampler (G1329B), and diode array detector (G1315B, DAD). Briefly, the column was a Zorbax Eclipse plus C18 (250 × 4.6 mm, 5 µm; Agilent); the column temperature was set to 30 °C and the flow rate was 0.8 mL·min^−1^. The injection volume was 10 μL, and the detection wavelengths were set to 280, 330, and 350 nm. Elution was carried out with a binary mobile phase of solvent A (water and 0.1% trifluoroacetic acid) and solvent B (acetonitrile). Data were processed using the Agilent OpenLAB CDS Chem-Station edition 2012. Molecule identifications were achieved as a function of the retention time of available standards, which were selected from the literature concerning their phenolic composition and their UV absorption spectra, as well as by adding standard solutions to the sample [55]. Quantification of individual phenolic compounds was performed using the external standard method curve, obtained with five concentration increments for each standard. The calibration curves for each standard solution, the limit of detection (LOD) and the limit of quantification (LOQ) are reported in Table 9. The curves exhibit a correlation coefficient above 0.9958. LOD and LOQ were established through calibration curves data based on the standard deviation (S) of the response at low concentration and the slope (σ): LOD = 3 S/σ and LOQ = 10 S/σ. LOD varied from 0.016 μg/mL (0.002 mg g^−1^ DW of gallic acid) to 0.087 μg/mL (0.003 mg g^−1^ DW of isorhamnetin 3-rutinoside). LOQ varied from 0.054 μg/mL (0.0015 mg g^−1^ DW of gallic acid) to 0.290 μg/mL (0.008 mg g^−1^ DW of isorhamnetin 3-rutinoside).

### 4.4. Data Analyses

The results of chemical determinations carried out on pears samples were subjected to a one-way analysis of variance, using Statgraphics Centurion XVI version [56] to test the effect of different cultivars on the following variables: concentrations on total phenolics, total flavonoids, antioxidant capacity, and individual phenolic compounds. Differences between means were assessed using Fisher’s least significant difference (LSD) procedure for means separation. The significance level was fixed at *p* ≤ 0.05 for all the statistical analyses.

## 5. Conclusions

The present study provides new insights regarding antioxidant capacity, total and individual phenolic compounds in fruit fractions (flesh, peel, core and peduncle) of Sardinian pear germplasm.

The investigated cultivars differed in the examined traits. In all cultivars, the peel and peduncle exhibited the highest content of bioactive compounds and antioxidant capacity. The cultivars Buttiru and Camusina had the highest values. Chlorogenic acid was the major individual phenolic compound in peel, flesh, and core, whereas arbutin dominated in the peduncle as the individual phenolic compound with the highest content.

Taken together, the results supplied novel information, which is essential for revising target valorisations of local pear cultivars and exploiting new promising sources of nutraceuticals.

Based on the results of the present research and on the novel queries, future investigation will focus on the nutritional and healthy properties of wild pear fruit to establish the effective benefits in terms of feed supply and ecosystem services attained by grafting with ancient cultivars.

## Figures and Tables

**Table 1:** Total antioxidant capacity (TEAC) by ABTS and DPPH methods, total phenolics (TotP), non-tannic phenolics (NTP), tannic phenolics (TP), total flavonoids (TotF), and condensed tannins (CT) in the fruit flesh of the investigated pear cultivars.

	TEAC		TotP	NTP	TP	TotF	CT
	(mmol 100 g^−1^ DM)		(g GAE kg^−1^ DM)			(g CE kg^−1^ DM)	(g DE kg^−1^ DM)
	ABTS	DPPH					
Camusina	1.5 ± 0.20 b	1.2 ± 0.02 b	8.9 ± 0.09 b	5.9 ± 0.36 ab	3.1 ± 0.46 b	1.2 ± 0.08 b	4.3 ± 0.12 b
Buttiru	2.7 ± 0.10 a	2.4 ± 0.09 a	17.7 ± 0.32 a	6.1 ± 0.78 a	11.5 ± 0.53 a	2.7 ± 0.06 a	8.2 ± 0.03 a
Spadona	0.9 ± 0.10 c	0.9 ± 0.06 c	6.7 ± 0.19 c	4.6 ± 0.12 bc	2.1 ± 0.26 b	0.9 ± 0.01 c	3.6 ± 0.36 c
Coscia	0.7 ± 0.06 c	0.8 ± 0.07 c	6.4 ± 0.28 c	4.2 ± 0.21 c	2.2 ± 0.23 b	0.6 ± 0.04 d	1.6 ± 0.01 d
Mean	1.4	1.3	9.9	5.2	4.7	7.4	4.4

In the columns, means followed by the same letter are not significantly different at *p* ≤ 0.05. Results are expressed as the mean ± SE (*n* = 3).

**Table 2:** Total antioxidant capacity (TEAC) by ABTS and DPPH methods, total phenolics (TotP), non-tannic phenolics (NTP), tannic phenolics (TP), total flavonoids (TotF), and condensed tannins (CT) in the fruit peel of the investigated pear cultivars.

	TEAC		TotP	NTP	TP	TotF	CT
	(mmol 100 g^−1^ DM)		(g GAE kg^−1^ DM)			(g CE kg^−1^ DM)	(g DE kg^−1^ DM)
	ABTS	DPPH					
Camusina	6.4 ± 0.22 a	5.9 ± 0.29 a	40.3 ± 0.73 a	15.1 ± 0.08 a	25.3 ± 0.81 a	7.2 ± 0.16 a	6.0 ± 0.03 b
Buttiru	5.5 ± 0.25 b	6.1 ± 0.55 a	36.3 ± 1.19 b	9.9 ± 0.75 bc	26.3 ± 0.86 a	6.2 ± 0.14 b	8.7 ± 0.42 a
Spadona	2.5 ± 0.06 c	2.1 ± 0.07 b	16.5 ± 0.52 c	8.9 ± 0.28 c	7.7 ± 0.61 b	2.4 ± 0.13 c	1.2 ± 0.06 c
Coscia	2.8 ± 0.17 c	2.5 ± 0.12 b	18.9 ± 0.27 c	11.5 ± 0.67 b	7.4 ± 0.94 b	2.6 ± 0.14 c	1.9 ± 0.01 c
Mean	4.3	4.2	28.0	11.4	16.7	4.6	4.4

In the columns, means followed by the same letter are not significantly different at *p* ≤ 0.05. Results are expressed as the mean ± SE (*n* = 3).

**Table 3:** Total antioxidant capacity (TEAC) by ABTS and DPPH methods, total phenolics (TotP), non-tannic phenolics (NTP), tannic phenolics (TP), total flavonoids (TotF), and condensed tannins (CT) in the fruit core of the investigated pear cultivars.

	TEAC		TotP	NTP	TP	TotF	CT
	(mmol 100 g^−1^ DM)		(g GAE kg^−1^ DM)			(g CE kg^−1^ DM)	(g DE kg^−1^ DM)
	ABTS	DPPH					
Camusina	5.8 ± 0.11 b	5.3 ± 0.09 b	34.9 ± 1.21 b	8.9 ± 0.14 c	26.0 ± 1.15 b	7.0 ± 0.22 b	12.0 ± 0.44 b
Buttiru	7.0 ± 0.05 a	6.8 ± 0.10 a	40.4 ± 0.59 a	11.6 ± 0.65 a	28.8 ± 0.06 a	8.6 ± 0.06 a	15.2 ± 0.51 a
Spadona	2.6 ± 0.15 d	2.1 ± 0.05 d	18.3 ± 0.34 d	8.2 ± 0.18 c	10.0 ± 0.18 d	2.9 ± 0.06 d	6.5 ± 0.15 d
Coscia	4.4 ± 0.05 c	3.4 ± 0.03 c	27.2 ± 0.66 c	10.2 ± 0.17 b	17.0 ± 0.52 c	4.1 ± 0.09 c	10.2 ± 0.03 c
Mean	5.0	4.4	30.2	9.7	20.5	5.7	10.1

In the columns, means followed by the same letter are not significantly different at *p* ≤ 0.05. Results are expressed as the mean ± SE (*n* = 3).

**Table 4:** Total antioxidant capacity (TEAC) by ABTS and DPPH methods, total phenolics (TotP), non-tannic phenolics (NTP), tannic phenolics (TP), total flavonoids (TotF), and condensed tannins (CT) in the fruit peduncle of the investigated pear cultivars.

	TEAC		TotP	NTP	TP	TotF	CT
	(mmol 100 g^−1^ DM)		(g GAE kg^−1^ DM)			(g CE kg^−1^ DM)	(g DE kg^−1^ DM)
	ABTS	DPPH					
Camusina	9.9 ± 0.41 ab	7.9 ± 0.13 a	58.8 ± 2.29 a	26.9 ± 0.43 a	32.0 ± 2.03 b	10.5 ± 0.12 a	7.0 ± 0.05 a
Buttiru	10.4 ± 0.05 a	8.1 ± 0.33 a	56.8 ± 0.85 a	20.5 ± 0.35 b	36.2 ± 1.00 a	11.3 ± 0.52 a	5.3 ± 0.14 b
Spadona	9.2 ± 0.23 bc	5.6 ± 0.09 b	42.2 ± 0.10 b	21.0 ± 0.05 b	20.5 ± 0.71 d	7.8 ± 0.08 b	4.2 ± 0.06 c
Coscia	8.8 ± 0.09 c	5.9 ± 0.27 b	45.4 ± 0.54 b	20.2 ± 0.21 b	25.3 ± 0.42 c	8.3 ± 0.08 b	4.2 ± 0.37 c
Mean	9.6	6.9	50.8	22.2	28.5	9.5	5.2

In the columns, means followed by the same letter are not significantly different at *p* ≤ 0.05. Results are expressed as the mean ± SE (*n* = 3).

**Table 5:** HPLC analysis of individual phenolic compounds (mg g^−1^ DM) in the fruit flesh of the investigated pear cultivars.

Phenolic Compounds	t_R_ * (min)	λmax ** (nm)	Camusina	Buttiru	Spadona	Coscia
Arbutin	5.08	280	0.21 ± 0.02 a	0.06 ± 0.001 d	0.09 ± 0.01 c	0.16 ± 0.001 b
Gallic acid	6.50	280	≤LOQ	≤LOQ	0.01 ± 0.0003	≤LOQ
Chlorogenic acid	10.69	330	0.67 ± 0.02 a	0.57 ± 0.06 ab	0.48 ± 0.04 b	0.50 ± 0.05 b
Catechin	11.40	280	0.04 ± 0.001	0.12 ± 0.001	nd	≤LOQ
Epicatechin	13.06	280	0.07 ± 0.001	0.07 ± 0.001	nd	nd
Sum			0.99	0.82	0.58	0.66

t_R_ * = retention time; λmax ** = wavelengths of maximum absorption in the UV region. In the rows, means followed by the same letter are not significantly different at *p* ≤ 0.05. nd = not detected; LOQ = limit of quantification. Results are expressed as the mean ± SE (*n* = 3).

**Table 6:** HPLC analysis of individual phenolic compounds (mg g^−1^ DM) in the fruit peel of the investigated pear cultivars.

Phenolic Compounds	t_R_ * (min)	λmax ** (nm)	Camusina	Buttiru	Spadona	Coscia
Arbutin	5.08	280	1.71 ± 0.10 a	0.12 ± 0.004 d	0.46 ± 0.01 c	0.85 ± 0.02 b
Gallic acid	6.50	280	0.02 ± 0.0006 a	≤LOQ	0.02 ± 0.001 a	0.01 ± 0.0003 b
Chlorogenic acid	10.69	330	1.34 ± 0.03 a	0.61 ± 0.05 b	0.32 ± 0.02 c	1.28 ± 0.05 a
Catechin	11.4	280	0.26 ± 0.02 b	0.37 ± 0.01 a	0.03 ± 0.0003 c	0.03 ± 0.004 c
Epicatechin	13.06	280	0.58 ± 0.01 a	0.35 ± 0.01 b	0.11 ± 0.01 c	0.07 ± 0.004 d
Rutin	20.4	350	0.37 ± 0.008 a	0.22 ± 0.002 b	0.16 ± 0.004 c	0.24 ± 0.006 b
Quercetin 3-galattoside	20.90	350	0.37 ± 0.002 a	0.38 ± 0.004 a	0.12 ± 0.003 b	0.05 ± 0.004 c
Quercetin 3-glucoside	21.4	350	nd	nd	0.25 ± 0.02	nd
Quercetin 3-*O*-(6″-*O*-malonyl)-β-glucoside	22.3	350	0.03 ± 0.001 a	0.04 ± 0.001 a	≤LOQ	≤LOQ
Isorhamnetin derivative	22.87	350	0.83 ± 0.028 a	0.52 ± 0.004 b	0.09 ± 0.002 d	0.20 ± 0.0004 c
Isorhamnetin 3-rutinoside	23.09	350	0.62 ± 0.036 a	0.45 ± 0.011 b	0.14 ± 0.002 c	0.18 ± 0.005 c
Isorhamnetin derivative	23.4	350	0.21 ± 0.004 a	0.09 ± 0.001 d	0.17 ± 0.011 b	0.11 ± 0.002 c
Isorhamnetin derivative	23.97	350	0.17 ± 0.005 b	0.20 ± 0.005 a	0.11 ± 0.001 c	0.05 ± 0.001 d
Isorhamnetin derivative	24.4	350	0.16 ± 0.007 c	0.29 ± 0.001 a	0.23 ± 0.002 b	0.06 ± 0.001 d
Sum			6.67	3.64	2.21	3.13

t_R_ * = retention time; λmax ** = wavelengths of maximum absorption in the UV region. In the rows, means followed by the same letter are not significantly different at *p* ≤ 0.05. nd = not detected; LOQ = limit of quantification. Results are expressed as the mean ± SE (*n* = 3).

**Table 7:** HPLC analysis of individual phenolic compounds (mg g^−1^ DM) in the fruit core of the investigated pear cultivars.

Phenolic Compounds	t_R_ * (min)	λmax ** (nm)	Camusina	Buttiru	Spadona	Coscia
Arbutin	5.08	280	1.35 ± 0.07 a	1.16 ± 0.02 b	0.81 ± 0.02 c	1.29 ± 0.08 ab
Gallic acid	6.50	280	0.02 ± 0.0004 a	0.02 ± 0.0002 a	0.01 ± 0.0008 b	≤LOQ
Chlorogenic acid	10.69	330	1.61 ± 0.001 a	1.26 ± 0.067 b	1.23 ± 0.029 b	0.65 ± 0.009 c
Catechin	11.4	280	0.23 ± 0.011 b	0.61 ± 0.003 a	0.12 ± 0.008 c	0.21 ± 0.008 b
Epicatechin	13.06	280	0.22 ± 0.006 b	0.41 ± 0.035 a	0.09 ± 0.001 c	0.12 ± 0.003 c
Di-*O*-caffeolylquinic acid	23.5	330	0.15 ± 0.001 a	0.06 ± 0.002 b	0.04 ± 0.0004 c	0.06 ± 0.004 b
Flavanol	31.9	280	nd	4.91 ± 0.02	nd	nd
Flavanol	39	280	0.22 ± 0.0006 b	0.36 ± 0.0006 a	0.17 ± 0.003 d	0.18 ± 0.006 c
Sum			3.80	8.79	2.47	2.51

t_R_ * = retention time; λmax ** = wavelengths of maximum absorption in the UV region. In the rows, means followed by the same letter are not significantly different at *p* ≤ 0.05. nd = not detected; LOQ = limit of quantification. Results are expressed as the mean ± SE (*n* = 3).

**Table 8:** HPLC analysis of individual phenolic compounds (mg g^−1^ DM) in the fruit peduncle of the investigated pear cultivars.

Phenolic Compounds	t_R_ * (min)	λmax ** (nm)	Camusina	Buttiru	Spadona	Coscia
Arbutin	5.08	280	7.09 ± 0.28 a	5.11 ± 0.05 c	7.49 ± 0.31 a	6.19 ± 0.19 b
Gallic acid	6.50	280	0.03 ± 0.001 b	0.03 ± 0.001 b	0.05 ± 0.005 a	0.03 ± 0.001 b
Chlorogenic acid	10.69	330	2.64 ± 0.04 a	2.02 ± 0.05 c	2.26 ± 0.05 b	2.55 ± 0.11 a
Catechin	11.4	280	0.27 ± 0.002 b	0.39 ± 0.008 a	0.22 ± 0.015 c	0.20 ± 0.011 c
Epicatechin	13.06	280	0.58 ± 0.01 a	0.52 ± 0.02 a	0.29 ± 0.01 b	0.57 ± 0.03 a
Rutin	20.4	350	0.28 ± 0.015 a	0.11 ± 0.003 c	0.05 ± 0.001 d	0.19 ± 0.005 b
Quercetin 3-galattoside	20.90	350	0.31 ± 0.008 a	0.18 ± 0.001 b	0.04 ± 0.001 d	0.06 ± 0.002 c
Quercetin 3-glucoside	21.4	350	0.48 ± 0.010 a	0.33 ± 0.012 b	0.05 ± 0.002 d	0.11 ± 0.003 c
3,5-Di-*O*-caffeoylquinic acid	22.2	330	0.042 ± 0.001 b	0.043 ± 0.001 b	0.051 ± 0.001 a	0.021 ± 0.001 c
Quercetin 3-*O*-(6″-*O*-malonyl)-β-glucoside	22.3	350	0.03 ± 0.001 a	0.03 ± 0.001 a	≤LOQ	≤LOQ
Di-*O*-caffeolylquinic acid	23.5	330	1.26 ± 0.02 a	0.94 ± 0.01 b	0.47 ± 0.01 c	0.38 ± 0.01 d
Di-*O*-caffeolylquinic acid	24.7	330	0.11 ± 0.002 b	0.17 ± 0.004 a	0.099 ± 0.003 c	0.039 ± 0.001 d
Flavanol	39	280	0.28 ± 0.003 a	0.23 ± 0.001 c	0.16 ± 0.002 d	0.25 ± 0.004 b
Sum			13.40	10.10	11.23	10.59

t_R_ * = retention time; λmax ** = wavelengths of maximum absorption in the UV region. In the rows, means followed by the same letter are not significantly different at *p* ≤ 0.05. nd = not detected; LOQ = limit of quantification. Results are expressed as the mean ± SE (*n* = 3).

**Table 9:** Chromatographic parameters of the quantitative evaluation of the phenolic compounds.

Phenolic Compounds	Linearity Range (µg mL^−1^)	Calibration Curves	LoD (µg mL^−1^)	LoQ (µg mL^−1^)	R^2^
Arbutin	2.5–300	Y = 3.2145x − 0.739	0.071	0.237	0.9997
Chlorogenic acid	0.75–50	Y = 32.335x − 51.97	0.027	0.092	0.9958
Gallic acid	1.5–6	Y = 18.314x	0.016	0.054	0.9998
Catechin	1–20	Y = 7.664x − 1.1724	0.029	0.099	0.9992
Rutin	0.3–20	Y = 17.359x + 3.5614	0.017	0.057	0.9991
Epicatechin	2.5–30	Y = 8.1588x − 2.5825	0.073	0.245	0.9967
Quercetin 3-galattoside	5–50	Y = 27.129x − 8.2984	0.033	0.110	0.9998
Quercetin 3-glucoside	0.2–20	Y = 23.502x − 7.2335	0.038	0.127	0.9992
Quercetin 3-*O*-(6″-*O*-malonyl)-β-glucoside	0.5–10	Y = 12.63 − 1.8529	0.071	0.2375	0.9999
Isorhamnetin 3-rutinoside	1–30	Y = 17.258x − 9.3267	0.087	0.290	0.9987
3,5-Di-*O*-caffeoylquinic acid	0.6–30	Y = 23.741x − 7.5373	0.050	0.168	0.9994

## Data Availability

Not applicable.

## References

[1] Azzini E., Maiani G., Durazzo A., Foddai M.S., Intorre F., Venneria E., Polito A.S. (2019). Giovanni varieties (*Pyrus communis* L.): Antioxidant properties and phytochemical characteristics. Oxid. Med. Cell. Longev..

[2] Ghazouani T., Talbi W., Sassi C.B., Fattouch S. (2020). Pears. Nutritional Composition and Antioxidant Properties of Fruits and Vegetables.

[3] Reiland H., Slavin J. (2015). Systematic review of pears and health. Nutr. Today.

[4] Johnson S.A., Navaei N., Pourafshar S., Akhavan N.S., Elam M.L., Foley E., Clark E.A., Payton M.E., Arjmandi B.H. (2016). Fresh pear (*Pyrus communis*) consumption may improve blood pressure in middle-aged men and women with metabolic syndrome. FASEB J..

[5] Navaei N., Pourafshar S., Akhavan N.S., Foley E.M., Litwin N.S., George K.S., Hartley S.C., Elam M.L., Rao S., Arjmandi B.H. (2017). Effects of Fresh Pear Consumption on Biomarkers of Cardiometabolic Health in Middle-Aged and Older Adults with Metabolic Syndrome. FASEB J..

[6] Hu D., Huang J., Wang Y., Zhang D., Qu Y. (2014). Fruits and vegetables consumption and risk of stroke: A meta-analysis of prospective cohort studies. Stroke.

[7] Meccariello R., D’Angelo S. (2021). Impact of polyphenolic-food on longevity: An elixir of life. An overview. Antioxidants.

[8] Hong S.Y., Lansky E., Kang S.S., Yang M. (2021). A review of pears (*Pyrus* spp.), ancient functional food for modern times. BMC Complement. Med. Ther..

[9] Akagić A., Oras A., Gaši F., Meland M., Drkenda P., Memić S., Hudina M. (2022). A comparative study of ten pear (*Pyrus communis* L.) cultivars in relation to the content of sugars, organic acids, and polyphenol compounds. Foods.

[10] Öztürk A., Demirsoy L., Demirsoy H., Asan A., Gül O. (2015). Phenolic compounds and chemical characteristics of pears (*Pyrus Communis* L.). Int. J. Food Prop..

[11] Li X., Li X., Wang T., Gao W. (2016). Nutritional composition of pear cultivars (*Pyrus* spp.). Nutritional Composition of Fruit Cultivars.

[12] Pes G.M., Dore M.P., Tsofliou F., Poulain M. (2022). Diet and longevity in the Blue Zones: A set-and-forget issue?. Maturitas.

[13] Puddu G., Falcucci A., Maiorano L. (2012). Forest changes over a century in Sardinia: Implications for conservation in a Mediterranean hotspot. Agrofor. Syst..

[14] Fois M., Bacchetta G., Cogoni D., Fenu G. (2018). Current and future effectiveness of the Natura 2000 network for protecting plant species in Sardinia: A nice and complex strategy in its raw state?. J. Environ. Plan. Manag..

[15] Sulas L., Re G.A., D’hallewin G. (2019). Agroforestry & Transumance in Sardinia.

[16] Agabbio M., Ansaldi N., Beccaro G.L., Berra L., Botta R., Brunu A. (2015). Frutti dimenticati e biodiversità recuperata. Il germoplasma frutticolo e viticolo delle agriculture tradizionali italiane. Casi di studio: Piemonte e Sardegna. Quad. Nat. Biodivers..

[17] Sau S., Pastore C., D’hallewin G., Dondini L., Bacchetta G. (2020). Characterisation of microsatellite loci in Sardinian pears (*Pyrus communis* L. and *P. spinosa* Forssk.). Sci. Hortic..

[18] Loru L., D’hallewin G., Satta A., Sulas L., Molinu M.G., Piluzza G., Pusceddu M., Pantaleoni R.A. FOR[m]AGE, BEES & FRUITS: Bee-fruit synergies with forage farming systems in rainfed Mediterranean environment. Agroforestry for the transition towards sustainability and bioeconomy, P3.3-5_216. Proceedings of the 5th European Agroforestry Conference.

[19] Agabbio M., D’Aquino S., Piga A. Risposta alla frigoconservazione di alcune cultivar di pere estive del germoplasma autoctono. Proceedings of the Atti del 2 Convegno Nazionale Biodiversità e Produzioni Biologiche.

[20] D’Aquino S., Agabbio M., Molinu M.G., Petretto A., Rosas G. Varietà di pero a maturazione autunnale e invernale del germoplasma autoctono: Aspetti qualitativi e fisiologia postraccolta. Proceedings of the Atti del 4° Convegno Nazionale Biodiversità Germoplasma Locale e Sua Valorizzazione.

[21] Manzoor M., Anwar F., Bhatti I.A., Jamil A. (2013). Variation of phenolics and antioxidant activity between peel and pulp parts of pear (*Pyrus communis* L.) fruit. Pak. J. Bot..

[22] Du B., Cheng C., Chen Y., Wu J., Zhu F., Yang Y., Peng F. (2021). Phenolic profiles and antioxidant activities of exocarp, endocarp, and hypanthium of three pear cultivars grown in China. J. Food Bioact..

[23] Liaudanskas M., Zymonė K., Viškelis J., Klevinskas A., Janulis V. (2017). Determination of the phenolic composition and antioxidant activity of pear extracts. J. Chem..

[24] Wang Z., Barrow C.J., Dunshea F.R., Suleria H.A.R. (2021). A Comparative Investigation on Phenolic Composition, Characterization and Antioxidant Potentials of Five Different Australian Grown Pear Varieties. Antioxidant.

[25] Suleria H.A., Barrow C.J., Dunshea F.R. (2020). Screening and characterization of phenolic compounds and their antioxidant capacity in different fruit peels. Foods.

[26] Jiang H., Wu F., Jiang X., Pu Y.F., Shen L.R., Wu C.Y., Bai H.J. (2022). Antioxidative, cytoprotective and whitening activities of fragrant pear fruits at different growth stages. Front. Nutr..

[27] Hameed A., Liu Z., Wu H., Zhong B., Ciborowski M., Suleria H.A.R. (2022). A Comparative and Comprehensive Characterization of Polyphenols of Selected Fruits from the Rosaceae Family. Metabolites.

[28] Decros G., Baldet P., Beauvoit B., Stevens R., Flandin A., Colombié S., Gibon Y., Pétriacq P. (2019). Get the balance right: ROS homeostasis and redox signalling in fruit. Front. Plant Sci..

[29] Kschonsek J., Wolfram T., Stöckl A., Böhm V. (2018). Polyphenolic compounds analysis of old and new apple cultivars and contribution of polyphenolic profile to the in vitro antioxidant capacity. Antioxidants.

[30] Pascoalino L.A., Reis F.S., Prieto M.A., Barreira J.C., Ferreira I.C., Barros L. (2021). Valorization of bio-residues from the processing of main Portuguese fruit crops: From discarded waste to health promoting compounds. Molecules.

[31] Rabetafika H.N., Bchir B., Blecker C., Paquot M., Wathelet B. (2014). Comparative study of alkaline extraction process of hemicelluloses from pear pomace. Biomass Bioenergy.

[32] Yan L., Li T., Liu C., Zheng L. (2019). Effects of high hydrostatic pressure and superfine grinding treatment on physicochemical/functional properties of pear pomace and chemical composition of its soluble dietary fibre. LWT.

[33] Malhi N., Carragher J., Saarela M., Pahl S. (2021). A Review of Opportunities to Recover Value from Apple and Pear Pomace.

[34] Fernandes A., Simões S., Ferreira I.M., Alegria M.J., Mateus N., Raymundo A., de Freitas V. (2022). Upcycling Rocha do Oeste Pear Pomace as a Sustainable Food Ingredient: Composition, Rheological Behavior and Microstructure Alone and Combined with Yeast Protein Extract. Molecules.

[35] Sad T.G., Djafaridze I., Kalandia A., Vanidze M., Smilkov K., Jacob C. (2021). Antioxidant Properties of the Native Khechechuri Pear from Western Georgia. Science.

[36] Lomba-Viana X., Raymundo A., Prista C., Alegria M.J., Sousa I. (2022). Clean Label “Rocha” Pear (*Pyrus communis* L.) Snack Containing Juice by-Products and Euglena gracilis Microalgae. Front. Nutr..

[37] Hudina M., Orazem P., Jakopic J., Stampar F. (2014). The phenolic content and its involvement in the graft incompatibility process of various pear rootstocks (*Pyrus communis* L.). J. Plant Physiol..

[38] Kumar M., Barbhai M.D., Hasan M., Punia S., Dhumal S., Radha, Rais N., Chandran D., Pandiselvam R., Kothakota A. (2022). Onion (*Allium cepa* L.) peels: A review on bioactive compounds and biomedical activities. Biomed. Pharmacother..

[39] Veluchamy R.S., Mary R., Puthiya P.S.B., Pandiselvam R., Padmanabhan S., Sathyan N., Shil S., Niral V., Ramarathinam M.M., Lokesha A.N. (2023). Physicochemical characterization and fatty acid profiles of testa oils from various coconut (*Cocos nucifera* L.) genotypes. J. Sci. Food Agric..

[40] Salkić B., Cvrk R., Imširović E., Salkić A., Salkić E. (2020). Comparison of pomological and chemical properties of autochthonous pear varieties with standard pear varieties. Int. J. Plant Soil Sci..

[41] Brahem M., Renard C.M., Eder S., Loonis M., Ouni R., Mars M., Le Bourvellec C. (2017). Characterization and quantification of fruit phenolic compounds of European and Tunisian pear cultivars. Food Res. Int..

[42] Kolniak-Ostek J., Oszmiański J. (2015). Characterization of phenolic compounds in different anatomical pear (*Pyrus communis* L.) parts by ultra-performance liquid chromatography photodiode detector-quadrupole/time of flight-mass spectrometry (UPLC-PDA-Q/TOF-MS). Int. J. Mass Spectrum..

[43] Cui T., Nakamura K., Ma L., Li J.Z., Kayahara H. (2005). Analyses of arbutin and chlorogenic acid, the major phenolic constituents in oriental pear. J. Agric. Food Chem..

[44] Bacchiocca M., Biagiotti E., Ninfali P. (2006). Nutritional and technological reasons for evaluating the antioxidant capacity of vegetable products. Ital. J. Food Sci..

[45] Lin L.Z., Harnly J.M. (2008). Phenolic compounds and chromatographic profiles of pear skins (*Pyrus* spp.). J. Agric. Food Chem..

[46] Azzini E., Durazzo A., Polito A., Veneria E., Foddai M.S., Zaccaria M., Mauro B., Intorre F., Maiani G. (2012). Biodiversity and local food products in Italy. Sustain. Diets Biodivers..

[47] Kolniak-Ostek J. (2016). Content of bioactive compounds and antioxidant capacity in skin tissues of pear. J. Funct. Foods.

[48] Hudina M., Stampar F., Orazem P., Petkovsek M.M., Veberic R. (2012). Phenolic compounds profile, carbohydrates and external fruit quality of the ‘Concorde’pear (*Pyrus communis* L.) after bagging. Can. J. Plant Sci..

[49] Galvis-Sanchez A.C., Gill-Izquierdo A., Gill M.I. (2003). Comparative study of six pear cultivars in terms of their phenolic and vitamin C contents and antioxidant capacity. J. Sci. Food Agric..

[50] Takebayashi J., Ishii R., Chen J., Matsumoto T., Ishimi Y., Tai A. (2010). Reassessment of antioxidant activity of arbutin: Multifaceted evaluation using five antioxidant assay systems. Free Radic. Res..

[51] Dell’Arca A.M. (2000). Agricoltura di Sardegna.

[52] Molinu M.G., Sulas L., Campesi G., Re G.A., Sanna F., Piluzza G. (2023). Subterranean Clover and Sulla as Valuable and Complementary Sources of Bioactive Compounds for Rainfed Mediterranean Farming Systems. Plants.

[53] Sanna F., Piluzza G., Campesi G., Molinu M.G., Re G.A., Sulas L. (2022). Antioxidant Contents in a Mediterranean Population of *Plantago lanceolata* L. Exploited for Quarry Reclamation Interventions. Plants.

[54] Re G.A., Piluzza G., Sanna F., Molinu M.G., Sulas L. (2019). Polyphenolic composition and antioxidant capacity of legume-based swards are affected by light intensity in a Mediterranean agroforestry system. J. Sci. Food Agric..

[55] Molinu M.G., Piluzza G., Campesi G., Sulas L., Re G.A. (2019). Antioxidant sources from leaves of Russian dandelion. Chem. Biodivers..

[56] StatPoint Technologies Inc (2009). Statgraphics Centurion XVI. User Manual.

